# Submissive Behavior and Cyber Bullying: A Study on the Mediator Roles of Cyber Victimization and Moral Disengagement

**DOI:** 10.5334/pb.509

**Published:** 2020-01-03

**Authors:** Bahtiyar Eraslan-Çapan, Fuad Bakioğlu

**Affiliations:** 1 Anadolu University, Institute of Education Sciences, Eskişehir, TR; 2 Karamanoğlu Mehmetbey University, Faculty of Education, Karaman, TR

**Keywords:** Submissive behavior, Cyber bullying, Cyber victimization, Moral disengagement, Adolescents

## Abstract

In order to prevent cyberbullying and cyber-victim behaviors that are very common among adolescents, it is important to investigate the factors that underlie these behaviors. The purpose of the present study was to examine the mediator roles of cyber victimization and moral disengagement in the relationship between submissive behavior and cyber bullying. The participants involved 370 Turkish adolescents (female: 47%; male, 53%). The age of participants ranged between 12 and 19 years (M = 15.92, SD = 1.87). Data were collected using the Submissive Behavior Scale, the Cyber Bullying Scale, the Cyber Victimization Scale, and the Moral Disengagement Scale. The data were analyzed using structural equation modeling. A bootstrapping analysis was conducted in order to determine any indirect effects. Structural equation modeling results provided evidence of indirect effects of submissive behavior on cyber bullying mediated by cyber victimization and moral disengagement. Bootstrapping showed that submissive behavior exerted a significant indirect effect on cyber bullying via cyber victimization and moral disengagement. The findings emphasized the role of youth cyber victimization and moral disengagement in explaining the relationship between submissive behavior and cyber bullying. The results of the study were discussed based on relevant literature, and suggestions for future studies were made.

## Introduction

In the last three decades, parallel to technological development, traditional aggression behaviors have become widespread as cyber bullying behaviors. Cyber bullying is a common problem that affects cyber victims in Turkey as well as in the whole world, especially in adolescence. It is observed that studies have mostly focused on cyber bullying behaviors and the factors affecting the cyber victimization behaviors have been emphasized less. Moreover, there are also limited number of studies in which cyber bullying and cyber victimization are involved and mediator factors are examined. In this study, the interrelation of cyber victimization and cyber bullying behaviors and the role of mediating personality traits were investigated and the findings were considered to be used for preventive efforts.

### Cyber bullying and cyber victimization

In electronic bullying research, cyber bullying and cyber victimization are treated as interoperable processes (Hood & Duffy, 2018). Cyber bullying is defined as intentional and repetitive bullying behaviors aimed at harming the victim by means of electronic texts that are different from traditional bullying and cyber victimization is defined as being exposed to such behaviors (Patchin & Hinduja, 2006; Piotrowski, 2012). While cyber bullying and cyber victimization behaviors have been observed from primary school to university life, it is stated that this kind of behavior peaks especially between the ages of 11–15 (Tokunaga, 2010) and 11–16 (Smith et al., 2008).

Although it is stated that cyber bullying and cyber victimization rates are quite high in adolescents, it is common for cyber bullying/victimization behaviors to be seen in the same individuals. In studies on adolescents, 70% (Johnson, 2015), 67% (Eroğlu et al., 2015), 29.5% (Mishna et al., 2012), 23.8% (Arıcak et al., 2008), 21.1% (Erdur-Baker, 2010) 8.9% (Sabancı, 2018), 6.2% (Evegü, 2014), and 7% (Kowalski, Limber, & Agaston, 2012) of individuals were reported to be both bullies and victims.

### Consequences of cyberbullying and cyber victimization

The fact that cyber bullying and cyber victimization are frequently experienced especially in adolescence and the negativities created by both conditions increase the severity of the situation. In comparison to pure cyberbullies or pure cyber victims, bully/victims have been found to suffer the most adverse consequences of cyberbullying in regards to their psychological and physical health, suicidal ideation, and academic performance (Kowalski & Limber, 2013; Bonanno & Hymel, 2013). It is observed that cyber bullying adolescents violate the rules, have a hostile attitude towards individuals around them (Arıcak et al., 2008), experience psychological incompatibility (Çetin, et al., 2012), and demonstrate aggression (Ybarra & Mitchell, 2007) while the cyber victims experience disappointment, sorrow (Raskauskas & Stoltz, 2007), anger, anxiety, academic motivation loss, academic failure, absenteeism (Beran & Li, 2005), and suicidal problems (Hinduja & Patchin, 2009) and their wellbeing and life quality decreases (Blais, 2008). In a focus-group study, adolescents defined cyber bullying as a situation which “is constant all the time, really hard to escape, you haven’t got friends around you to support you, loads of people can see it if it’s on the internet” (Smith et al., 2008), which is important in terms of understanding adolescents’ cyberbullying and cyber-victim behaviors and shows the severity of adolescents’ adverse perceptions. Since cyber bullying can take place at any time during the day or at night, the behaviors can be demonstrated anonymously, they can reach a large number of people quickly through a large number of channels, the victim cannot escape the effects of this behavior and have the potential to cause further damage (Moses, 2013), cyberbullying-victimization behaviors are seen as important to determine the relevant variables and take precautions.

### Cyber victimization and cyber bullying Relationships

Cyber bullying/cyber victimization rates of adolescents are quite high not only in Turkey but also in the world. This situation shows that adolescents have the potential to be cyber bullies as well as cyber victims. It can be explained by the interrelation between cyber bullying and cyber victimization (Wong, Chan, & Cheng, 2014) and the most powerful determinant of cyber bullying is being cyber victim (Hood & Duffy, 2018; Johnson, 2015; Kowalski et al., 2014; Kwan & Skoric, 2013). That is, individuals who cyberbully others also tend to be victims of cyberbullying (cyber bully/victims). This situation can be explained through Kowalski et al’s (2014) model focusing on the transition from cyberbullying to cyber victimization. According to the model, individuals’ personal characteristics and situational factors might cause cyber victimization. These cyber victimization experiences affect individuals’ ideas, emotions, and stimulation and have them evaluate their scenario and decide on how to respond to bullying experiences. Some cyber victims might respond by aggressive and cyberbullying behaviors. According to this model, socially inadequate personality traits cause individuals to become cyber-victims and personality traits such as moral disengagement are effective in making decisions to respond to the situation in by cyber-bullying behaviors (Johnson, 2015; Kowalski et al., 2014).

This situation is explained by the desire of individuals exposed to cyber bullying to do the same harm to others and the feeling of revenge, and this leads to cyber bullying or violence (Bauman, Toomey, & Walker, 2013; Dioguardi & Theodore, 2006; Yaman & Peker, 2012).

### The role of submissive personality trait in cyber victimization

Submissive personality trait refers to acting in accordance with the rules and orders issued by the authority and changing or being obliged to change the individual’s thoughts, convictions or value judgments in the direction the dominant authority wants (Budak, 2003). It is observed that adolescents who exhibit submissive behaviors have low self-esteem and cannot stand up for their own rights due to their insufficient social abilities, are open to the manipulation of others and are prone to cyber victimization due to these characteristics (Modecki et al., 2013). The studies showed that submissive behaviors explained 36% of cyber victimization (Peker, Eroğlu & Çitemel, 2012), submissive adolescents had passive, obedient, anxious, sensitive, insecure, and cautious traits (Peker, Eroğlu & Çitemel, 2012), and submissive adolescents became cyber victims by demonstrating passive and obedient behaviors against the aggressive behaviors towards them (Dioguardi & Theodore, 2006). Moreover, it is seen that submissive individuals have lower self-esteem and those with lower self-esteem may ‘attract’ victimization because they communicate, verbally or non-verbally, that they will not defend themselves, or they may fail to defend themselves when victimized, thus increasing the likelihood of repeated victimization (Van Geel, et al., 2018). In short, it is considered that adolescents with submissive personality traits will be prone to cyber-victimization due to their passive personality.

### The role of moral disengagement during the transition from cyber victimization to cyberbullying

In the literature, it is indicated that cyber victims responding aggressively to the experience of victimization and becoming cyberbullies (Johnson, 2015). Moreover, there are studies reporting the important role of personality traits such as moral disengagement during the transition from cyber victimization to cyberbullying (George, 2014; Hood & Duffy, 2018; Johnson, 2015; Kowalski et al., 2014). Morally justifying the behavior by moving away from moral responsibilities and creating moral justifications to harm victims without experiencing guilt or conscience is defined as moral disengagement (Bandura, 1999). Moral disengagement mechanisms reduce the expected negative effects of negative behaviors by cognitive restructuring of harmful behaviors, reducing personal responsibility for harmful behavior, ignoring the consequences of harmful behavior, and seeing the victim as inhuman (Bandura, 1991). As a recent concept, moral disengagement strategies were determined to be closely associated with cyber bullying and cyber victimization (Hood & Duffy, 2018; Moses, 2013; Perren et al., 2012; Pornari & Wood, 2010; Postorino, 2014). Additionally, it was emphasized that moral disengagement was the most powerful risk factor and higher moral disengagement strengthened the cyber victimization-bullying relationship (Hood & Duffy, 2018). Adolescents that were exposed to cyberbullying could demonstrate the same behaviors considering that those cyberbullying them deserved the similar behaviors that were experienced by themselves (Johnson, 2015; Kowalski et al., 2014). The studies show that individuals who suffered from cyber victimization show revenge drive after a while and display cyber bullying behaviors in order to make others experience what they experienced (Bauman, Toomey, & Walker, 2013; Dioguardi & Theodore, 2006; Yaman & Peker, 2012).

Adolescents who suffer from cyber bullying demonstrate these behaviors towards others and use moral disengagement strategies to preserve their self-concept and conscience (Kowalski et al., 2014). Therefore, adolescents who suffer from cyber victimization use their moral disengagement strategies by loosening their internal self-regulatory mechanisms to justify their harmful and aggressive behaviors. Research on the issue emphasized that decreasing moral disengagement strategies would diminish especially cyberbullying behaviors of cyber victims (Hood & Duffy, 2018). In an experimental study aimed at preventing bullying, the program focusing on moral disengagement behaviors was found to reduce the bullying and victim behaviors of adolescents (Wang & Goldberg, 2017).

### Present study

In this study, the mediator role of personality traits in the transition from cyber victimization to cyberbullying. The study was based on the theoretical framework proposed by Kowalski et al (2014) on the transition from cyber victimization to cyberbullying. Based on this theoretical model, it was assumed that submissive personality traits involving inadequate social skills and obedient behaviors would lead to cyber victimization and cyber victims would respond to bullying with cyberbullying by demonstrating moral disengagement behaviors.

**Figure 1:**
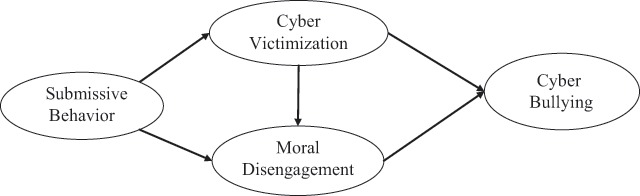
The hypothesized structural model.

As a result, it is seen that there is an important problem that should be intervened urgently in schools because of the permanent and negative effects of cyber bullying/cyber victimization behaviors and a limited number of studies on the behaviors that turn from cyber victimization to cyberbullying was conducted. In these limited number of studies, it has been revealed that individuals with submissive personality trait (lacking required skills to stand up for their rights) were open to become cyber victims (Atik, Özmen & Kemer, 2012), individuals who had been victims considered that what they experienced was deserved by others as well (Johnson, 2015) and demonstrated submissive behaviors could have a more hostile attitude (Diguardi & Theodore, 2006), thus, they could use moral disengagement strategies to take revenge (George, 2014; Hood & Duffy, 2018; Johnson, 2015; Kowalski et al., 2014) and had a potential to become cyber bullies. Therefore, the purpose of this study was to investigate the relationship between submissive personality traits and cyber bullying through cyber victimization and moral disengagement strategies. The hypothesized model regarding this purpose can be seen in Figure 1.

## Method

### Participants

In this study, convenience sampling method was used. The sample of 370 volunteered adolescents from different middle and high school in the northwest part of Turkey was recruited between September 2017 and December 2017. The mean age of the participants was 15.92 years (Standard Deviation = 1.87) with a range from 12 to 19 years. Of these participants, 47% (N = 174) were female and 53% (N = 196) were male.

### Measures

The data for this study were collected using the Submissive Act Scale (Gilbert & Alan, 1994), the Cyber Bullying Inventory (Topçu & Erdur-Baker, 2010), and the Collective Moral Disengagement Scale (Gini et al., 2014). Detailed information concerning these measures has been presented below.

#### Submissive Behavior Scale

Submissive behavior was measured with the Submissive Behavior Scale (SBS) developed by Gilbert and Alan (1994). The SBS is a self-report questionnaire with 16 items. Items are rated on a 5-point Likert scale from 1 (never) to 5 (always). Items include statements such as “I let others criticize me or put me down without defending myself”. The total score of the Turkish-SBS was the sum of the 16 items ranging from 16 to 80, with higher scores indicating the submissive behavior level. SBS was translated into Turkish by Savaşır and Şahin (1997). The Turkish version of the SBS have good construct validity and internal reliability (Cronbach’s α = .89) and test-retest reliability (α = .84). In this study, the SBS also exhibited good reliability (Cronbach’s α = .74).

#### Cyber Bullying Inventory

Cyber bullying and cyber victimization were measured with the Cyber Bullying Inventory (CBI) developed by Topçu and Erdur-Baker (2010). CBI was composed of two subscales. These subscales were Cyber Bullying Scale (CBS) and Cyber Victimization scales (CVS). The CBS is a self-report questionnaire with 14 items. Items are rated on 4-point Likert scale from 1 (never) to 4 (more than three times). Items include statements such as “to send hurtful e-mails someone known”. The total score of the CBS was the sum of the 14 items ranging from 14 to 56, with higher scores indicating the cyber bullying level. CBS have good construct validity (RMSEA = .06, GFI = .93, AGFI = .89, CFI = .93, TLI = .90 and NFI = .89) and internal reliability (Cronbach’s α = .86) and test-retest reliability (α = .82). In this study, the CBS also exhibited excellent reliability (Cronbach’s α = .83). The CVS is a self-report questionnaire with 14 items. Items are rated on 4-point Likert scale from 1 (never) to 4 (more than three times). Items include statements such as “insulting the chatroom”. The total score of the CVS was the sum of the 14 items, ranging from 14 to 56 with higher scores indicating the cyber victimization level. CVS have good construct validity (RMSEA = .06, GFI = .93, AGFI = .90, CFI = .89, TLI = .86 and NFI = .84) and internal reliability (Cronbach’s α = .80) and test-retest reliability (α = .82). In this study, the CVS also exhibited good reliability (Cronbach’s α = .79).

#### Collective Moral Disengagement Scale

Moral disengagement was measured with the Collective Moral Disengagement Scale (CMDS) developed by Gini and his colleagues (2014). The CMDS consist of 15 moral disengagement behavior related items such as “little lies can be said if it doesn’t do any harm”. Each item is rated on a 5-point Likert-type scale, ranging between 1 (no one) and 5 (everyone). The total score of the CMDS was the sum of the 15 items ranging from 15 to 75, with higher scores indicating the moral disengagement level (Gini et al. 2014). CMDS was adapted into Turkish by Eraslan-Çapan and Bakioğlu (2016). The Turkish version of the CMDS have good construct validity (*χ*^2^/df = 3.20, RMSEA = .08, GFI = .89, AGFI = .85, CFI = .94, NNFI = .93 and SRMR = .06) and internal reliability (Cronbach’s α = .86) and test-retest reliability (α = .86). In this study, the CMDS also exhibited excellent reliability (Cronbach’s α = .83).

### Procedure

The participants completed paper-and-pencil questionnaires in a classroom environment. In the data collection stage of the research, the assessment tools were prepared as a leaflet and distributed to students in a classroom environment, all of whom had volunteered to participate in the research. Before each application, the researchers introduced themselves and explained the importance and purpose of the research. In addition, the researchers told the participants that there would be no individual evaluation and no requirement for identity information and that the results would be used for scientific purposes only. The participants were allowed to answer the questionnaires at their own pace and typically took about 20 minutes to complete all of the sections.

### Data Analysis

Descriptive analyzes of this study were performed using IBM SPSS Statistics 22.0. and the structural equation model and mediation model were performed in AMOS Graphics. Discriminant validity and internal consistency were conducted with MS Excel. We tested the structural model using maximum likelihood estimation. Item parceling method was used in order to reduce the number of observed variables and to improve reliability and normality of the resulting measures (Nasser-Abu Alhija & Wisenbaker, 2006). A parceling technique was used in order to avoid errors sourcing from one-dimensional measures. Besides, item parceling method allows us to control for inflated measurement errors due to multiple items for the latent variable (Little et al., 2002). Two, two, two and three parcels were obtained from the Submissive Behavior Scale, the Cyber Bullying Scale, the Cyber Victimization Scale and the Collective Moral Disengagement Scale, respectively.

Several indices of goodness-of-fit were used as criteria for the above model selection. We used *χ*^2^/df< 5, CFI, TLI, GFI, IFI >.90, SRMR and RMSEA <.08 as the assessment standards of the model fit index (Hu & Bentler, 1999; MacCallum et al., 1996; Tabachnick & Fidell, 2007). We performed bootstrapping tests of mediation to examine whether cyber victimization and moral disengagement mediated the relation between submissive behavior and cyber bullying (Preacher & Hayes, 2008). The Bootstrapping Confidence interval was estimated in the indirect impact of moral disengagement on cyber bullying. 10000 resampling and 95% confidence intervals were used in this process. Confidence intervals that do not contain zero indicate effects that are significant at .05.

## Results

### Descriptive Statistics

The total scores of all variables in this study are presented in terms of gender. The mean score obtained from submissive behavior (\bar X:31.06) was close to the mean score of both the female (\bar X:31.13) and the male participants (\bar X:31.00). The mean score obtained from cyber bullying (\bar X:16.16) was higher than the mean score of the female participants (\bar X:15.59), but lower than the score of the male participants (\bar X:16.67). Similarly, the mean score obtained from moral disengagement (\bar X:33.71) was higher than the mean score of the female participants (\bar X:30.26), but lower than score of the male participants (\bar X:36.76). The mean score obtained from cyber victimization (\bar X:16.74) was higher than the mean score of the female participants (\bar X:16.13), but lower than the score of the male participants (\bar X:17.29).

### Measurement Model and CFA

First, we tested the measurement model to assess whether each of the latent variables was represented by their indicators. The measurement model consisted of four latent factors, submissive behavior, cyber bullying, cyber victimization, and moral disengagement, and nine observed variables. The measurement model test indicated a satisfactory model fit: *χ*^2^_(21, N = 370)_ = 19.448, *p* < .001; *χ*^2^/df = .926; GFI = .99; CFI = 1.00; NFI = .99; TLI = 1.00; SRMR = .020; RMSEA = .001. The summary of the CFA is presented in Table 1.

**Table 1:** Summary of the CFA.

Variables	Factor Mean	Factor SD	Factor alpha	Composite reliability	AVE	Loading	Error

Submissive Behavior							
SBPar1	31.06	8.38	.74	.83	.70	.82	.32
SBPar2						.90	.20
Cyber Bullying							
CBPar1	16.16	3.09	.83	.90	.84	.92	.16
CBPar2							
Cyber Victimization							
CVPar1	16.74	3.10	.79	.77	.70	.82	.33
CVPar2						.86	.27
Moral Disengagement							
MDPar1	33.71	11.05	.83	.81	.74	.84	.29
MDPar2						.84	.30
MDPar3						.83	.31

*Note*: n = 370, explained variance 79.78%.

As presented in Table 1, each of the latent variables, number of items, internal consistency coefficient (≥0.70; Huck, 2012; Nunnally, 1978) and factor loads (i.e., ≥0.32; Worthington & Whittaker, 2006) were found to be adequate. The measurement model explained 79% of the total variance (≥50; Henson & Roberts, 2006). In addition, the convergent and discriminant validity analysis was conducted to see to what extent the variables present share the variances and how different they are from other measures (Fornell & Larcker, 1981). Moreover, the composite reliability coefficients (≥0.70; Nunnally, 1978) and average variance extracted coefficient (≥.50; AVE) were investigated. These values were found .70 and above. The factor loadings of all the indicators were significant (ranging from .82 to .92, *p* < .001), demonstrating that respective indicators were true representative of their latent factors.

### Preliminary Analyses

The relationships among submissive behavior, cyber bullying, cyber victimization, and moral disengagement were analyzed using structural equation modeling. The analysis was performed in two steps. In the first step, descriptive statistics were determined. In the second step, the hypothesized model was tested. The descriptive statistics between the associated variables are presented in Table 2.

**Table 2:** Correlations among the variables of interest.

Variable	1	2	3	4	5	6	7	8	9

1. SBPar1	(.68)								
2. SBPar2	.61**	(.80)							
3. CBPar1	.43**	.30**	(.84)						
4. CBPar2	.41**	.30**	.69**	(.84)					
5. CVPar1	.41**	.35**	.65**	.61**	(.67)				
6. CVPar2	.40**	.35**	.57**	.59**	.57**	(.73)			
7. MDPar1	.31**	.21**	.37**	.32**	.32**	.26**	(.71)		
8. MDPar2	.31**	.16**	.43**	.38**	.37**	.31**	.59**	(.69)	
9. MDPar3	.35**	.23**	.44**	.40**	.37**	.36**	62**	63**	(.68)
M	15.04	16.02	7.97	8.20	8.27	8.47	10.44	10.04	11.00
SD	4.62	4.72	1.66	1.71	1.71	1.82	3.93	3.99	3.98

*Note*: ** *p* < .01, SBPar submissive behavior parcels, CBPar cyber bullying parcels, CVPar cyber victimization parcels, MDPar moral disengagement parcels, M mean, SD standard deviation. AVE’s square root is in parentheses.

When Table 2 is examined, it can be seen that there is a significant positive correlation between submissive behavior parcels and cyber bullying parcels (*r* = .30 ≤ *r* ≤ .43, *p* < .01), between submissive behavior parcels and cyber victimization parcels (*r* = .35 ≤ *r* ≤ .41, *p* < .01) and between submissive behavior parcels and moral disengagement parcels (*r* = .16 ≤ *r* ≤ .35, *p* < .01). Moreover, there was a significant positive correlation between cyber victimization parcels and cyber bullying parcels (*r* = .57 ≤ *r* ≤ .65, *p* < .01) and between cyber victimization parcels and moral disengagement parcels (*r* = .26 ≤ *r* ≤ .37, *p* < .01). The square root of the AVE coefficients among the variables of the study ranged from .67 to .80.

### Mediation Analyses

**Figure 2:**
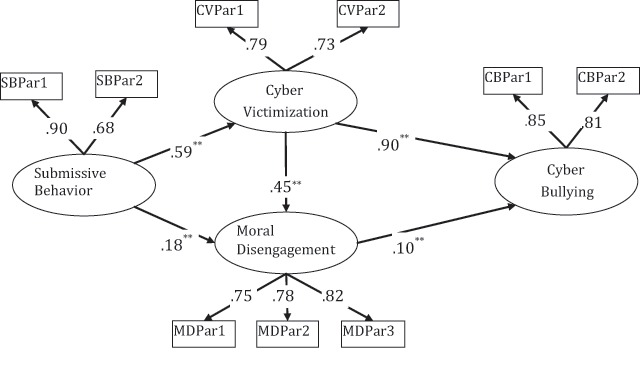
Mediation for submissive behavior on cyber bulling via cyber victimization and moral disengagement.

In the second step, the structural equation model was tested. In this step, it was determined whether cyber victimization and moral disengagement had a mediating role in the relationship between submissive behavior and cyber bullying. The analysis results are presented in Figure 2.

All path coefficients were observed to be significant in the analysis. Submissive behavior predicted cyber victimization positively (*β* = .59, *p* < .01) and moral disengagement positively (*β* = .18, *p* < .01). Cyber victimization predicted moral disengagement positively (*β* = .45, *p* < .01) and cyber bullying positively (*β* = .90, *p* < .01). In addition, moral disengagement predicted cyber bullying positively (*β* = .10, *p* < 0.01). Moreover, the effect coefficient of submissive behavior predicting cyber bullying through the mediation of cyber victimization and moral disengagement was estimated to be .57.

When the fit indexes of structural equation model are examined, it can be said that all values are acceptable levels. The fit indexes were as follows: *χ*^2^_(22, N = 370)_ = 20.193, *p* < .001; *χ*^2^/df = .918; GFI = .99; CFI = 1.00; NFI = .99; TLI = 1.00; SRMR = .021; RMSEA =.001. Therefore, it can be stated that the structural equation model was confirmed.

In the bootstrapping analysis, 10000 resampling methods were used to determine the significance of direct and indirect effects. The results of bootstrapping coefficients, 95% confidence interval upper and lower bounds are presented in Table 3.

**Table 3:** Bootstrapping results.

Model paths	Coefficient	95% C. I.	

		Lower bound	Upper bound

**Direct effect**			
Submissive Behavior →Cyber Victimization	.59	.45	.70
Submissive Behavior →Moral Disengagement	.18	.03	.35
Cyber Victimization →Moral Disengagement	.45	.29	.60
Cyber Victimization →Cyber bullying	.90	.79	.99
Moral Disengagement →Cyber Bullying	.10	.02	.22
**Indirect effect**			
Submissive Behavior →Cyber Victimization →Moral Disengagement →Cyber Victimization	.57	.44	.68

When Table 3 is summarized, all the effects in the structural equation model were significant. There are no zero values in the upper and lower bounds of the bootstrapping confidence interval for direct and indirect effects. In the light of these results, it can be said that the adolescents’ submissive behavior had an effect cyber bullying behaviors through the mediation of collective moral disengagement and cyber victimization behaviors.

## Discussion

With widespread and active use of the Internet, cyber bullying and cyber victimization have been a major problem in the world. Therefore, it will be helpful to reveal protective and risk factors of cyber bullying and cyber victimization. In this study, the mediator role of cyber victimization and moral disengagement in the relationship between submissive behavior and cyber bullying of Turkish adolescents was investigated. As expected, the results show that the cyber victimization and moral disengagement plays a mediator role in the relationship between submissive behavior and cyber bullying. Accordingly, moral disengagement was positively correlated with submissive behavior and cyber victimization, and submissive behavior positively predicted cyber victim. In short, it can be expressed that as the adolescents’ submissive behavior level increased, their moral disengagement, cyber victimization, and cyber bullying behaviors increased as well.

Studies are in parallel with the research findings. In studies on adolescents, it has been found that submissive behaviors were an important predictor and risk factor of cyber victimization and cyber bullying. (Atik, Özmen, & Kemer, 2012; Kowalski et al., 2014; Ogurlu & Sarıçam, 2018; Özkan & Özen, 2008; Peker, Eroğlu, & Çitemel, 2012). It has been found that adolescents who were unable to protect their own rights and behave according to others’ wills were kept on being exposed to negative behaviors by remaining passive rather than blocking individuals who were bullying themselves or taking necessary intervention and help behaviors (Peker, Eroğlu, & Çitemel, 2012). Moreover, it was asserted that these individuals had low self-esteem and more prone to become cyber victims (Brewer, & Kerslake, 2015). As it is seen, the finding that submissive behaviors leading cyber victimization were supported in our study.

The other finding of the study was the relationship among the moral disengagement strategies of adolescents, cyber victimization, and cyber bullying behaviors. The literature shows that high moral disengagement increased the relationship between cyber victimization and cyber bullying (Hood & Duffy, 2018, Johnson, 2015). This finding is supported by other studies concluding that individuals who became cyber victims started to think that the cyberbullies deserved aggression or cyberbullying behaviors were not that bad (Johnson, 2015). It is indicated that cyber victims might use moral disengagement strategies since they feel disappointment, sorrow (Raskauskas & Stoltz, 2007), anger (Beran & Li, 2005), suicidal feelings (Hinduja & Patchin, 2009), revenge feelings (Bauman, Toomey & Walker, 2013; Dioguardi & Theodore, 2006; Yaman & Peker, 2012), and thoughts that others deserve hostile behaviors (Diguardi & Theodore, 2006, Johnson, 2015). It was also found that cyber victims felt shame and revenge more (Dilber, 2013) and 72% of cyber bullies demonstrate harmful behaviors for revenge or retaliation (Mark & Ratliffe, 2011), which supported the findings of the current study. In their study, Mishna et al., (2012) found that some students were shy people who could not bullies or demonstrate aggression in real life, and that they committed cyber bullying behaviors to avenge what they experienced in real life by making use of the opportunity to disguise their identity in the virtual world, which also supported the findings of the current study. In short, adolescents who were victims of cyber bullying due to their passive and submissive personality traits commit cyber bullying behaviors in order to avenge the bullying they were exposed to and use moral disengagement strategies to justify their bullying behaviors.

As a result of the bootstrapping analysis, it was found that the relationships among all variables were significant. Firstly, the effect size obtained in submissive behavior predicted moral disengagement was found to be low (Cohen, 1988, Sawilowsky, 2003). Moreover, the value of effect size obtained from the cyberbullying predictions of moral disengagement was found to be low. These results showed that submissive behavior alone is not sufficient to explain moral disengagement behavior, and moral disengagement also explains low levels of cyber bullying. The upper limit of the magnitude of effect size, which is explained by the submissive behavior of moral disengagement and cyber bullying of moral disengagement, was found to be moderate. Cyber victimization of submissive behaviors, moral disengagement of cyber victimization and cyber bullying of cyber victimization were seen to have high effect size values (Cohen, 1988). Therefore, it can be stated for bigger samples that submissive behaviors predicted cyber victimization and moral disengagement, cyber victimization predicted moral disengagement and cyberbullying, and moral disengagement predicted cyberbullying directly. Moreover, it was found that the results of this study were confirmed in bigger samples, and moral disengagement and cyber victimization played a mediator role in the relationship between submissive behaviors and cyberbullying.

## Conclusion

In this study, it is important to reveal the mediator role of personality traits in the relationship between cyber victimization and cyberbullying because it supported the framework of Kowalski et al. (2014). it was found that cyber victimization and moral disengagement mediated the relationship between submissive personality trait and cyber bullying behaviors. An important finding in this study was that the relationship between cyber victimization and cyber bullying in adolescents was interrelation and that the submissive personality trait and moral disengagement behaviors played a role in this relationship. The interrelation relationship between cyber bullying and cyber victimization is a common problem that negatively affects the physical, psychological and academic life areas of adolescents. In the efforts to rule out this problem, prevention of cyber victimization depends on the prevention of cyber bullying through the efforts at schools, and the prevention of cyber victimization depends on the improvement of submissive and moral disengagement personality. Therefore, the focus should be on the personality traits that predispose individuals to cyber bullying and cyber victimization in the interventions aiming at disposing these problems. In this study, submissive personality trait and moral disengagement are risk factors for the circular relationship between adolescents’ cyber bullying and cyber victimization. For this reason, cyber bullying and cyber victimization prevention programs in schools should focus on activities to raise awareness of behaviors that push adolescents to cyber bullying, to ensure that they know and protect their rights, to obtain skills of acting effectively, and to increase the personal responsibility of their behaviors. Moreover, the relationship among the cognitive beliefs, moral thoughts and behaviors of cyber bullies and cyber victims should be focused as well. As a matter of fact, in an experimental study aiming at preventing bullying, programs focusing on moral disengagement behaviors were found to reduce cyber bullying and victimization behaviors of adolescents (Wang & Goldberg, 2017).

## Limitation

There are some limitations in this research. Firstly, the data may involve with social desirability bias because the variables examined in this study are obtained by self-report scales. Secondly, the data of this study cannot be generalized to all students in Turkey since it was not obtained from a large number of schools. In future studies, the findings can be examined by gender with the same variables. Structural equation modeling was used in this study. In future studies, it can be recommended use qualitative research methods or different research methods.
